# Supplementary material for: Proteomic Analysis of Salivary Extracellular Vesicles from COVID-19 Patients Reveals a Specific Anti-COVID-19 Response Protein Signature
Source: Int J Mol Sci. 2024 Mar 26;25(7):3704. doi: 10.3390/ijms25073704 (PMC11011897; doi:10.3390/ijms25073704)
Supplement: Supplementary file 1 [file ijms-25-03704-s001.zip › SUPP_FIG_Legends_110324.pdf]

SUPPLEMENTARY FIGURES LEGENDS

Supplementary Figure S1. Functional annotations and over-representation analysis of the top 10 most enriched KEGG pathways of the exclusive and differentially expressed proteins (≥ 2-fold change, $p < 0.05$) in the Covid-19 (+) group. (A, C, and E): Hierarchical clustering tree summarizing the correlation among significant pathways related to gene set according to KEGG pathway, GO Molecular function, and GO Biological process, respectively. Pathways with many shared proteins are clustered together. Bigger dots indicate more significant p-values. (B, D, and F). Pathway-terms interactome network generated after enrichment analysis (p-value <0.005, FDR Q-value <0.05) according to KEGG pathway, GO Molecular function and GO Biological process, respectively. Each node represents an enriched pathway term. The nodes are connected by a line, whose thickness reflects the percent of overlapping proteins, and the size of the node corresponds to the number of proteins.

Supplementary Figure S2. Functional annotations over-representations on analysis of the top 10 most enriched KEGG pathway of the exclusive and differentially expressed proteins (≥ 2-fold change, $p < 0.05$) in the HCC group. (A, C, and E): Hierarchical clustering tree summarizing the correlation among significant pathways related to gene set according to KEGG pathway, GO Molecular function, and GO Biological process, respectively. Pathways with many shared proteins are clustered together. Bigger dots indicate more significant p-values. (B, D, and F): Pathway-terms interactome network generated after enrichment analysis (p-value <0.005, FDR Q-value <0.05) according to KEGG pathway, GO Molecular function and GO Biological process, respectively. Each

node represents an enriched pathway term. The nodes are connected by a line, whose thickness reflects the percent of overlapping proteins, and the size of the node corresponds to the number of proteins.

Supplementary Figure S3. Functional enrichment analysis of exclusive and more abundant proteins for both COVID-19 (+) and HCC group. (A), (C), and (E) Functional annotations and over-representation analysis of top 20 most enriched KEGG pathways, GO Molecular functions and GO Biological processes, respectively, of the pooled exclusive and differentially expressed proteins (≥ 2-fold change, $p < 0.05$) from the COVID-19 (+) and HCC group. (B), (D), and (F). Hierarchical clustering tree summarizing the correlation among significant pathways related to gene set according to KEGG pathway, GO Molecular function, and GO Biological process, respectively. Pathways with many shared proteins are clustered together. Bigger dots indicate more significant p-values.

Supplementary Figure S4. PPI network analysis in the COVID-19 (+) group. The protein-protein interactome was constructed from 124 differentially expressed proteins (35 exclusive and 89 more abundant proteins). The network consisted of 102 nodes and 330 edges. The edges indicate both functional and physical protein associations. Line thickness indicates the strength of data support. Minimum required interaction score was set at 0.4 (medium confidence). Data visualization was conducted in the STRING database, available at: https://string-db.org, accessed on Feb 23, 2024.

Supplementary Figure S5. PPI network analysis in the HCC group. The protein-protein interactome was constructed and from 201 differentially expressed proteins (72 exclusive and 129 more abundant proteins). The network consisted of 195 nodes and 610 edges. The edges indicate both functional and physical protein associations. Line thickness indicates the strength of data support. Minimum required interaction score was set at 0.4 (medium confidence).

Data visualization was conducted in the STRING database, available at: https://string-db.org, accessed on Feb 23, 2024.
