# Supplementary figures and images for: Proteomic Analysis of Salivary Extracellular Vesicles from COVID-19 Patients Reveals a Specific Anti-COVID-19 Response Protein Signature
Source: Int J Mol Sci. 2024 Mar 26;25(7):3704. doi: 10.3390/ijms25073704 (PMC11011897; doi:10.3390/ijms25073704)

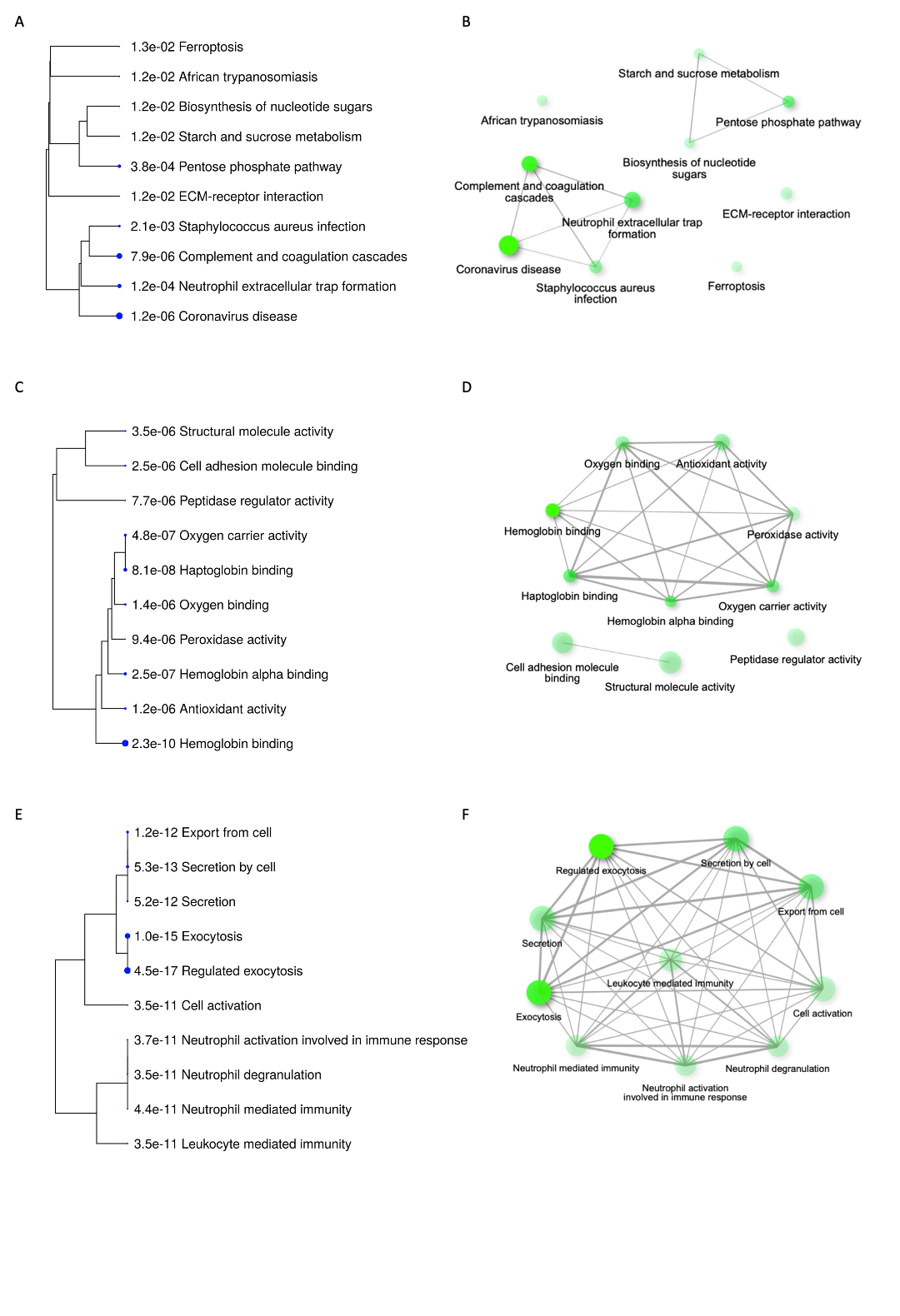

Supplement: Supplementary file 1 [file ijms-25-03704-s001.zip › Figure S1.tiff]

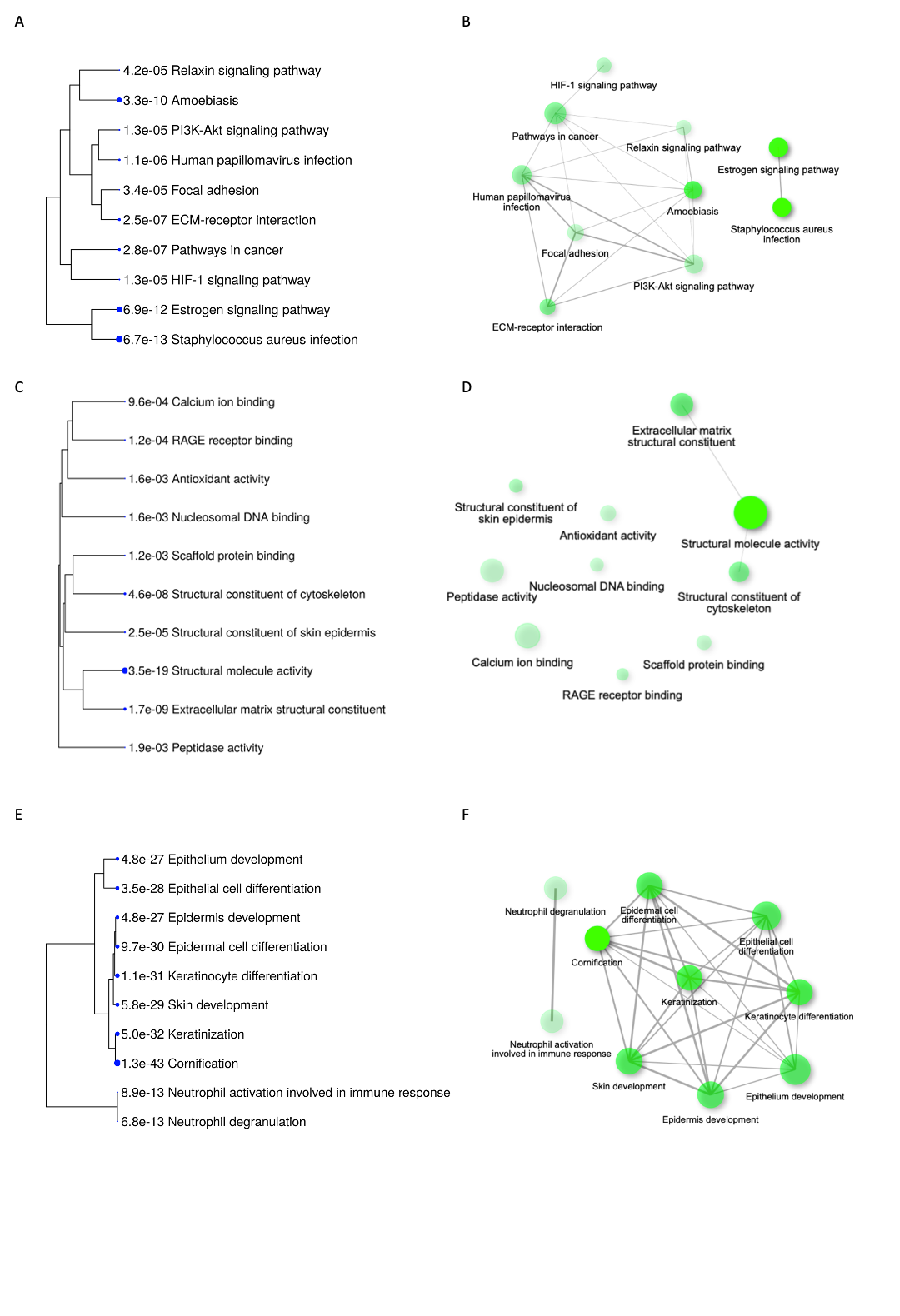

Supplement: Supplementary file 1 [file ijms-25-03704-s001.zip › Figure S2.tiff]

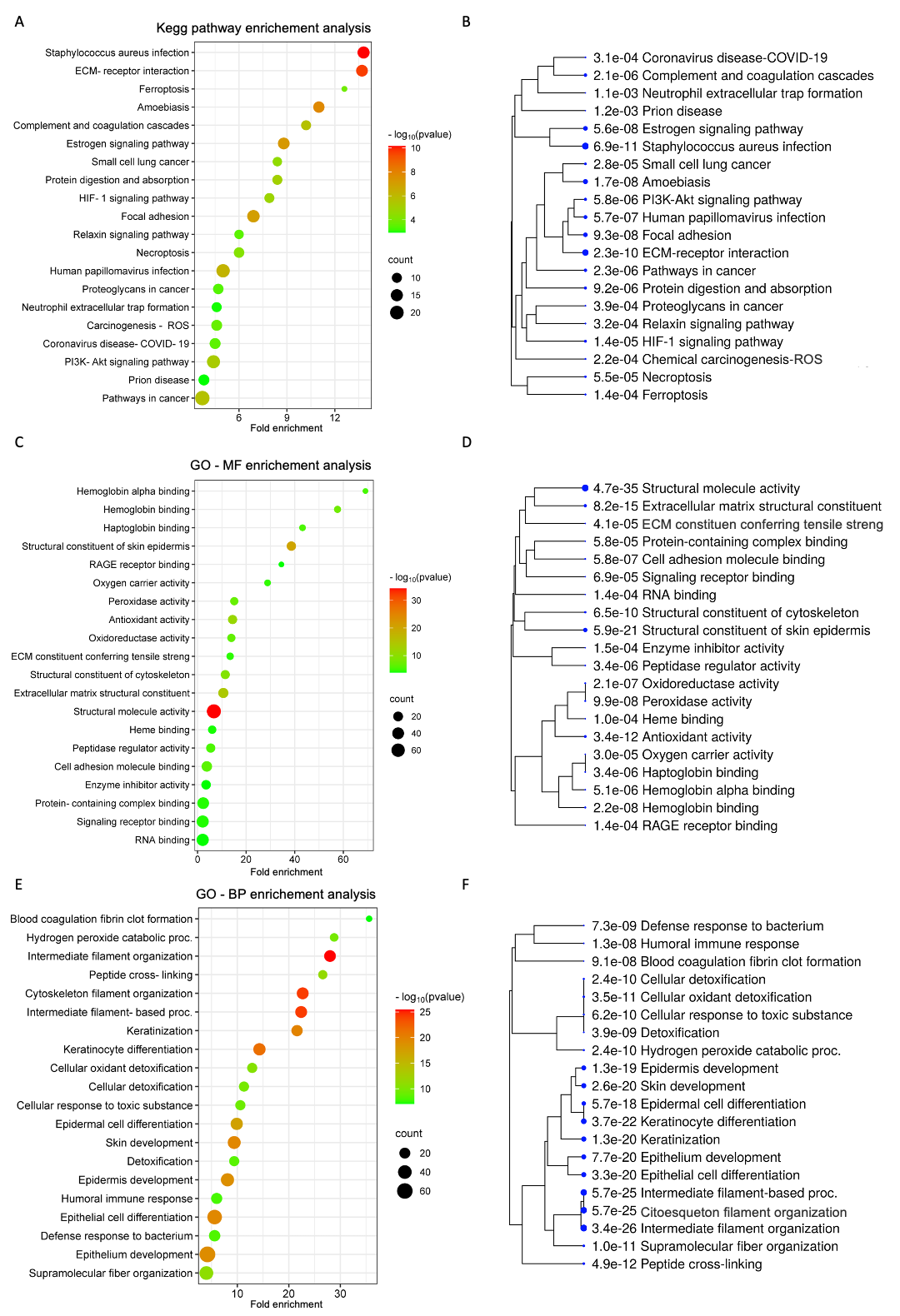

Supplement: Supplementary file 1 [file ijms-25-03704-s001.zip › Figure S3.tiff]

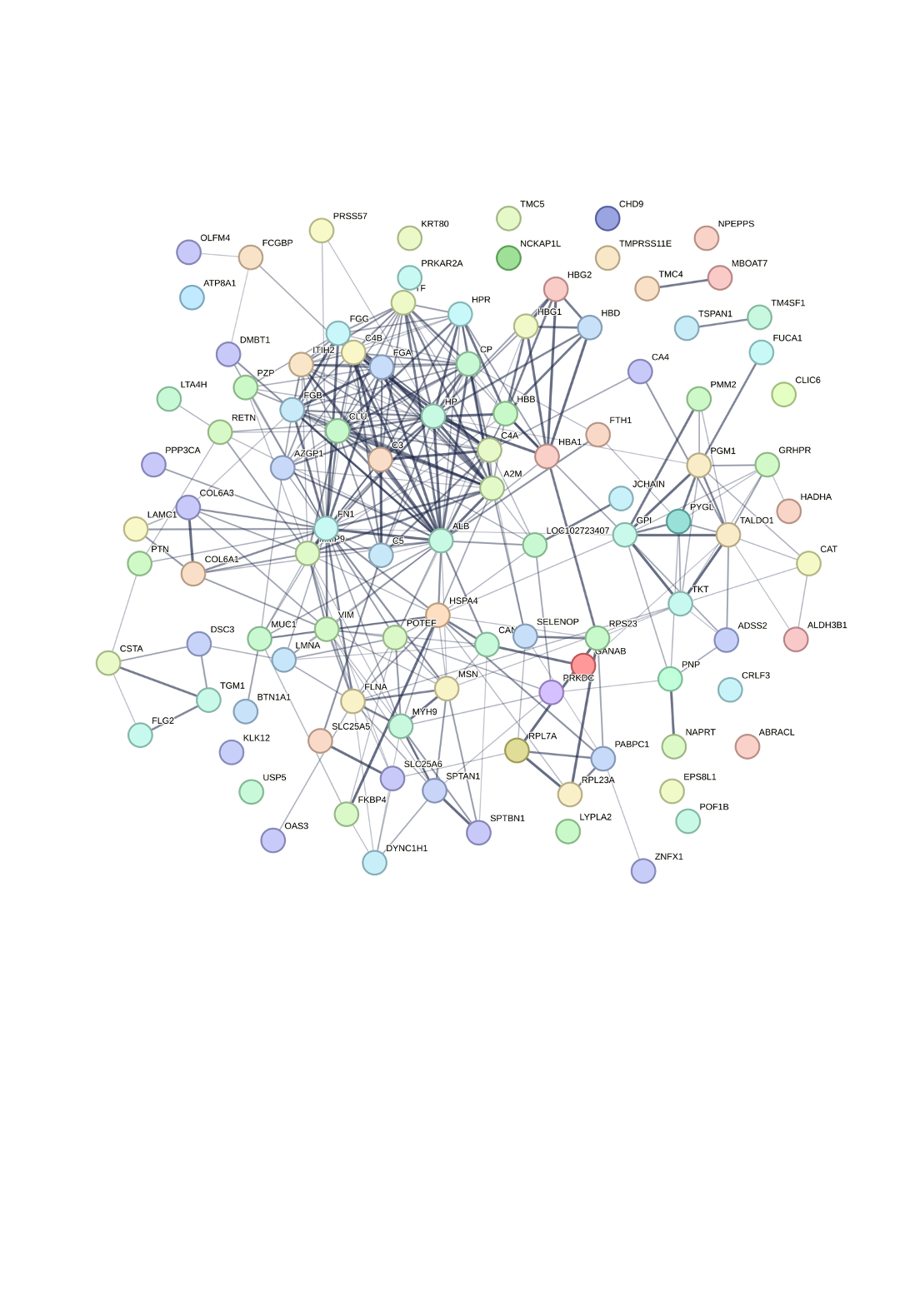

Supplement: Supplementary file 1 [file ijms-25-03704-s001.zip › Figure S4.tiff]

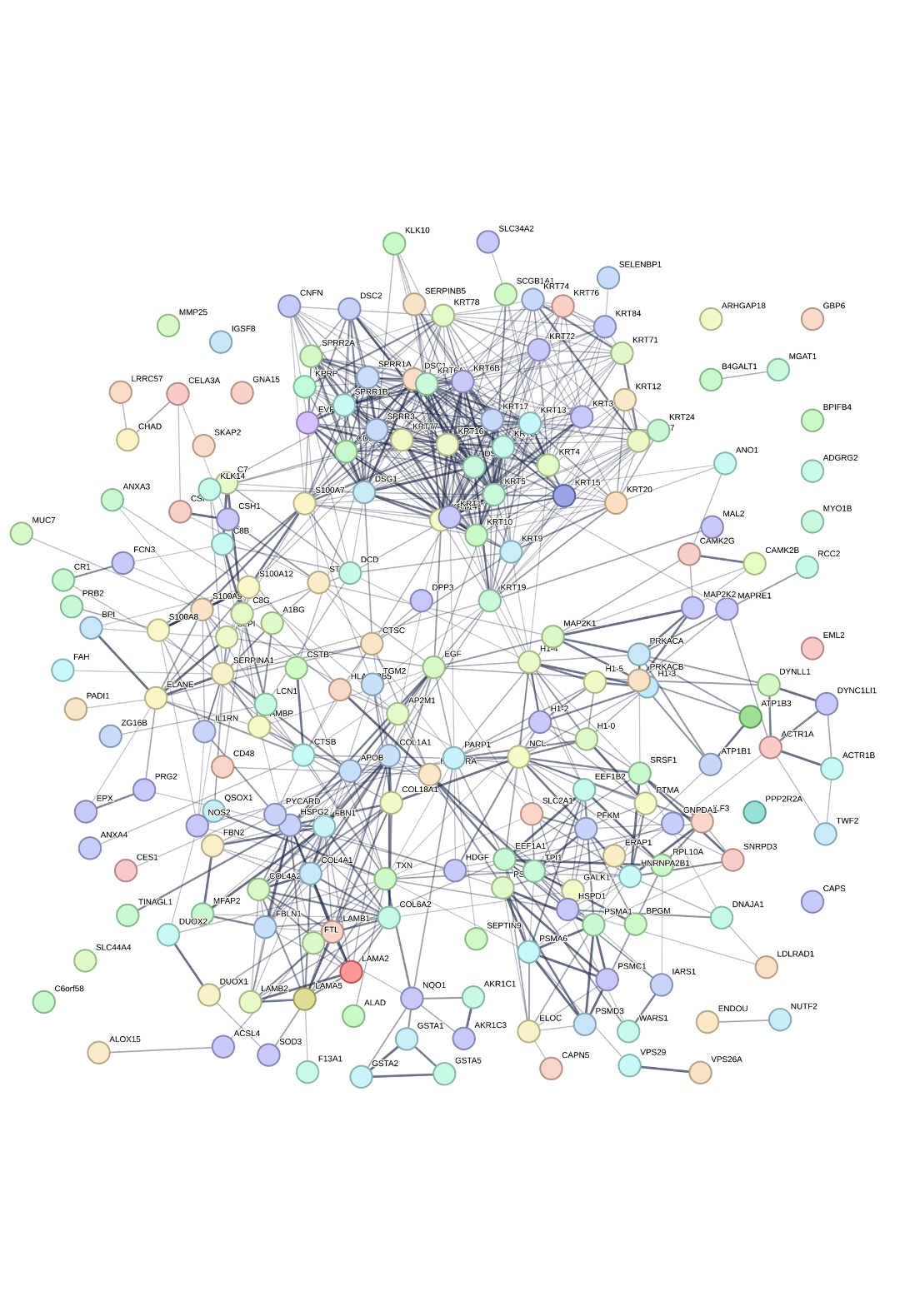

Supplement: Supplementary file 1 [file ijms-25-03704-s001.zip › Figure S5.tiff]
